# Image Pre-Processing Method of Machine Learning for Edge Detection with Image Signal Processor Enhancement

**DOI:** 10.3390/mi12010073

**Published:** 2021-01-11

**Authors:** Keumsun Park, Minah Chae, Jae Hyuk Cho

**Affiliations:** Department of Electronic Engineering, Soongsil University, Seoul 06978, Korea; sunsun@ssu.ac.kr (K.P.); minahchae@ssu.ac.kr (M.C.)

**Keywords:** CMOS image sensor, edge detection, machine learning, pre-process, image signal processor

## Abstract

Even though computer vision has been developing, edge detection is still one of the challenges in that field. It comes from the limitations of the complementary metal oxide semiconductor (CMOS) Image sensor used to collect the image data, and then image signal processor (ISP) is additionally required to understand the information received from each pixel and performs certain processing operations for edge detection. Even with/without ISP, as an output of hardware (camera, ISP), the original image is too raw to proceed edge detection image, because it can include extreme brightness and contrast, which is the key factor of image for edge detection. To reduce the onerousness, we propose a pre-processing method to obtain optimized brightness and contrast for improved edge detection. In the pre-processing, we extract meaningful features from image information and perform machine learning such as k-nearest neighbor (KNN), multilayer perceptron (MLP) and support vector machine (SVM) to obtain enhanced model by adjusting brightness and contrast. The comparison results of F1 score on edgy detection image of non-treated, pre-processed and pre-processed with machine learned are shown. The pre-processed with machine learned F1 result shows an average of 0.822, which is 2.7 times better results than the non-treated one. Eventually, the proposed pre-processing and machine learning method is proved as the essential method of pre-processing image from ISP in order to gain better edge detection image. In addition, if we go through the pre-processing method that we proposed, it is possible to more clearly and easily determine the object required when performing auto white balance (AWB) or auto exposure (AE) in the ISP. It helps to perform faster and more efficiently through the proactive ISP.

## 1. Introduction

After the invention of camera, the quality of image from machinery has been continuously improved and it is easy to access the image data. It is recognized as the main data itself and is used to extract additional information through complex data processing using artificial intelligence (AI) [1].

The CMOS Image Sensor is one of the microelectromechanical systems (MEMS) related image data expected to combine with different devices such as visible light communication (VLC), light detection and ranging (LiDAR), Optical ID tags, etc. With CMOS Image Sensor, image signal processor (ISP) treats attributes of image and produces an output image. However, traditional ISP system is not able to perfectly solve the problems such as detail loss, high noise and color rendering and not being appropriate for edge detection [2].

In image processing, edge detection is fundamentally important because they can quickly determine the boundaries of objects in an image [3]. Furthermore, edge detection is performed to simplify the image in order to minimize the amount of data to be processed. Moreover, computer vision technology has been developing, edge detection is considered essential for more challenging task such as object detection [4], object proposal [5] and image segmentation [6]. Therefore, it is necessary to develop suitable processor or method only for edge detection.

There are a variety of edge detection methods that are classified by different calculations and generates different error models. Prewitt, Canny, Sobel and Laplacian of Gaussian (LoG) are well-used operators of edge detection [7]. They are sensitive of noise so as to deal with the shortcomings, edge detection filters or soft computing approaches are introduced [8]. Computer vision technology can supplement deficiencies with machine learning. A lot of algorithms have been previously introduced to perform edge detection; gPb-UCM [9], CEDN [10], RCF [11], BDCN [12] and so on. As a part of these efforts, we propose pre-processing method to determine optimized contrast and brightness for edge detection with improved accuracy. We performed three types of machine learning models including MLP, SVM and KNN; all machine learning methods showed better F1 score than non-machine learned one, while pre-processing also scored better than non-treated one.

## 2. Materials and Methods

### 2.1. MEMS on Image Sensor and Processer

MEMS technology is used as a key sensor element required to the internet of things (IoT)-based smart home, innovative production system of smart factory, and plant safety vision system. In addition, intelligent sensors that are used in various fields, such as autonomous vehicles, robots, unmanned aerial vehicles and smartphones, where the smaller devices have more advantage. Accordingly, system-in-package (SiP) technology, which aggregates sensors and semiconductor circuits on one chip using MEMS technology, is used to develop intelligent sensors [13].

The CMOS image sensor can be mass-produced through the application of a logic large scale integration (LSI) manufacturing processor; it has the advantage of low manufacturing cost and low power consumption due to its small device size compared to a charge coupled device (CCD) image sensor having a high voltage analog circuit. With those factors driving the growth, the current image sensor market is expected to grow at an annual rate of about 8.6% from 2020 to 2025 to reach 28 billion in 2025 [14].

**Figure 1:**
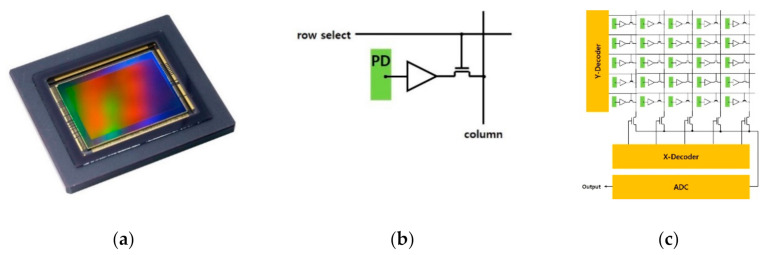
Complementary metal oxide semiconductor (CMOS) Image Sensor: (**a**) CMOS Sensor for industrial vision (Canon Inc., Tokyo, Japan); (**b**) Circuit of one pixel; (**c**) Pixel array and Analog Frontend (AFE).

A typical smart image sensor system implements the image-capturing device and the image processor into separate functional units: an array of pixel sensors and an off-array processing unit. A standard pixel array architecture includes the photodiode, gate switch, source follower and readout transistor. The reset gate resets the photodiode at the beginning of each capture phase. The source follower isolates the photodiode from the data bus. The analog signals from the sensor array take raw pixel values for further image processing as shown in Figure 1 [15].

**Figure 2:**
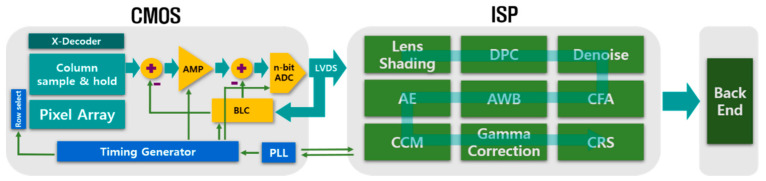
Conventional Structure of CMOS Image Sensor.

The ISP is a processing block that converts the raw digital image output from the AFE into an image that can be used for a given application. This processing is very complex and include a number of discrete processing blocks that can be arranged in a different order depending on the ISP [16]. ISP consists of Lens shading, Defective Pixel Correction (DPC), denoise, color filter array (CFA), auto white balance (AWB), auto exposure (AE), color correction matrix (CCM), Gamma correction, Chroma Resampler and so on as shown in Figure 2.

ISP has the information that can explain the image variation and computer vision can learn to compensate through that variation. Through this, computer vision can complement the function of ISP and if the function of ISP is used for low-level operations such as denosing, and computer vision is used for high-level operation; this can secure capacity and lower processing power [17].

Basic AE algorithms are a system which divides the image into five areas and place the main object on center, the background on top, and weights each area [18]. This approach is appropriate when the overall image is mid tone while proper exposure has not been performed with mixed contrast. To overcome this problem, study for judging the condition of the light source and auto selection of the method for targeted contrast. In detail, the algorithm terminates with normal contrast values between the background and object [19]. On the other hand, the algorithm continues when the state of light is backward or forwarded, compared to the average, and center values of the brightness levels of the entire image the illumination condition was divided into the brightness under sunshine and the darkness during night and according to each illumination condition experiment were performed with exposure, without exposure, and contrast stretch. As a result, when the image was with exposure, the edge detection was good and when the contrast stretch was performed, the edge detection value further increased [20].

### 2.2. Edge Detection

Edges are curves in which sudden changes in brightness or spatial derivatives of brightness occur [21]. Changes in brightness are where the surface direction changes discontinuously, where one object obscures another, where shadow lines appear or where the surface reflection properties are discontinuous. In each case, you need to find the discontinuity of the image brightness or its derivatives. Edge detection is a technique that produces pixels that are only on the border between areas and Laplacian of Gaussian (LoG), Prewitt, Sobel and Canny are widely used operators for edge detection. 

LoG uses the 2D Gaussian function to reduce noise and operate the Laplacian function to find the edge by performing second order differentiation in the horizontal and vertical directions [22].

Prewitt is used for vertical and horizontal edge detection. Compared to the Sobel mask, the edge comes out less but the speed is much faster. The operator uses two masks that provide detailed information about the edge direction when considering the characteristics of the data on the other side of the mask center point. The two masks are convolutional, with the original image to obtain separate approximations of the derivatives for the horizontal and vertical edge changes [23].

Sobel detects the amount of change by comparing each direction values based on the center using mask. It extracts vertical, horizontal and diagonal edges and is resistant to noise and as the mask gets bigger, the edges become thicker and sharper. However, change in contrast occurs frequently and is not effective in complex images [24]. A method of combining Sobel operator with soft-threshold wavelet denoising has also been proposed [25].

Canny edge detection is smoothed using a Gaussian filter to remove noise. After that, the size and direction are found using the gradient the maximum value of the edge is determined through the non-maximum suppression process and the last edge is classified through hysteresis edge tracking [26]. In recent research, a median filter was used instead of Gaussian filtering to reduce the effect of noise and remove isolated points [27].

We used canny because it has the advantages of improving signal to noise ratio and better detection specially in noise condition compared to other operators mentioned above [28].

### 2.3. Dataset

**Figure 3:**
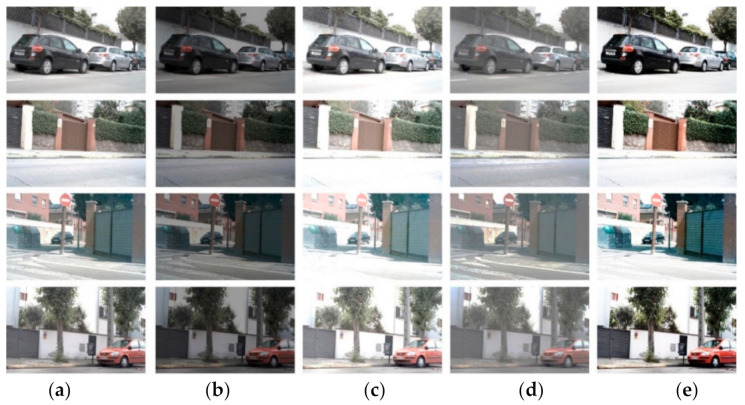
We augment input image data by putting differential in brightness and contrast using BIPED dataset. For more augmentation, it can be adjusted each and simultaneously on original image: (**a**) original image; (**b**) controlled image (darker); (**c**) controlled image (brighter); (**d**) controlled image (low contrast); (**e**) controlled image (high contrast).

Many works to make dataset for object and edge detection and image segmentation are known like BSDS500 [2] by Arbelaez et al., NYUD [29] by Silberman et al., Multicue [30] by Mely et al., BIPED [31] by Soria et al., etc. Although BSDS500 dataset, which is composed of 500 images for 200 training, 100 validation and 200 test images, is well-known in computer vision field, the ground truth (GT) of this dataset contains both the segmentation and boundary. BIPED, Barcelona Images for Perceptual Edge Detection, is a dataset with annotated thin edges. It is composed of 250 outdoor images of 1280 × 720 pixels and annotated by experts on the computer vision. This dataset is generated by the lack of edge detection datasets and available as a benchmark for evaluating edge detection. The dataset used in our study was performed using not only BIPED but also actual images taken using a camera of a Samsung Galaxy Note 9 driven by BSDS500 and CMOS image sensor. However, in the process of extracting the features of the histogram, BIPED was the most appropriate in the method mentioned above, so only BIPED was used. Using BIPED dataset, we carried out the image-transformation on brightness and contrast to augment the input image data as shown in Figure 3.

As BIPED has only 50 images for test data, we also need to increase the amount of them. Same task is applied to augment the test data.

### 2.4. Image Characteristics

Images are generated by the combination of an illumination source and reflection or absorption of energy from various elements of the scene being imaged [32]. We indicate images by two-dimensional functions of the form f (*x*, *y*). the value of f at spatial coordinates (*x*, *y*) is a scalar quantity that is characterized by two components: (*x*) is the amount of source illumination incident on the scene being viewed and (*y*) is the amount of illumination reflected by the objects in the scene. To interpret this information, we see an image histogram which is graphical representation of pixel intensity for the *x*-axis and number of pixels for *y*-axis. We analyze the histogram to extract the meaningful analysis for effective image processing.

We indicate images by two-dimensional functions of the form f (*x*, *y*). the value of f at spatial coordinates (*x*, *y*) is a scalar quantity that is characterized by two components: (*x*) is the amount of source illumination incident on the scene being viewed and (*y*) is the amount of illumination reflected by the objects in the scene. To interpret this information, we see an image histogram which is graphical representation of pixel intensity for the *x*-axis and number of pixels for *y*-axis. We analyze the histogram to extract the meaningful analysis for effective image processing.

We convert to RGB image data to grayscale and get the histogram. The *x*-axis has all available gray level from 0 to 255 and *y*-axis has the number of pixels that have a particular gray level value. We can get the information of brightness by observing the spatial distribution of the values. If the values are concentrated toward to the left, the image is darker. In contrast, if they are focused toward to the right, the image is lighter. Intensity levels is closely associated with the image contrast. Which is defined as the difference in intensity between the highest and lowest intensity levels in an image. When an appreciable number of pixels in an image have a high dynamic range, we typically expect the image to high contrast. Conversely, an image with low dynamic range especially the middle of the intensity scale indicates low contrast.

#### 2.4.1. Pixel Feature Normalization

**Figure 4:**
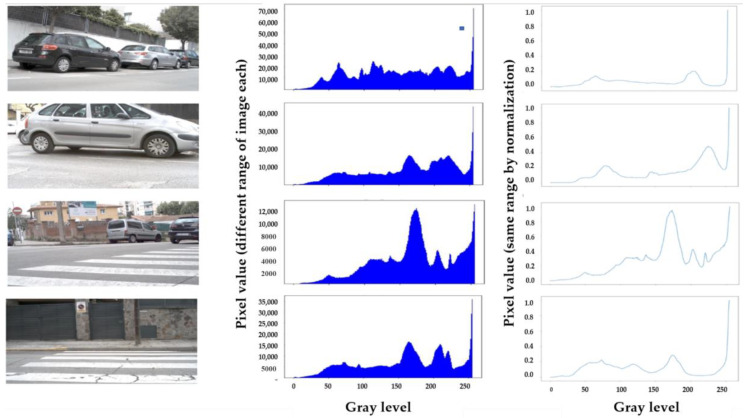
Example of normalization: (**a**) Original image; (**b**) Histogram of original image; (**c**) Normalized histogram of original image.

We did process for normalization, which is a process to view the meaningful data patterns or rules when data units do not match as shown in Figure 4. In most of applications, each image has a different range of pixel value, therefore normalization of the pixel is essential process of image processing. We need to transform features by scaling them to a given range between 0 and 1 by Min–Max-Scaler from sklearn.

#### 2.4.2. Histogram Information

**Figure 5:**
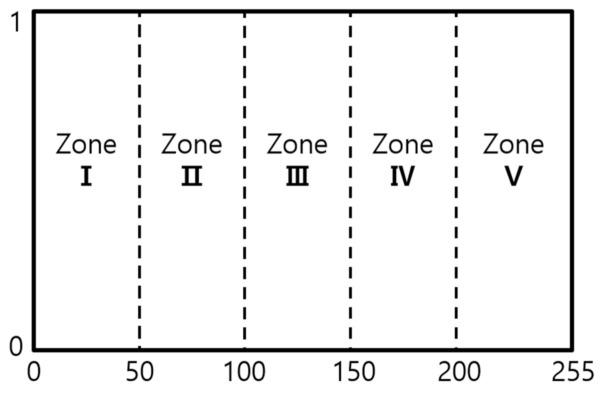
Definition of Zone in the normalized histogram of brightness.

To look through the characteristics of the training image, we investigated the histogram of image each. As shown in Table 1 and Figure 5, we categorize them into some distribution types of brightness and contrast according to concentration of peak, pixel intensity etc. In order to obtain the appropriate threshold in actual image with various illumination, it is estimated as an important task. The number of peaks and intensities is considered in divided zone of histogram, as shown in Figure 5. The intensity of each zone is scored as *I_zone_*, while the peak of each zone is scored as *P_zone_*, as follow,

$$I_{\mathrm{zone}}={\frac{{\mathrm{Intensity}}_{}{}_{\;}{\mathrm{of}}_{}{}_{\;}{\mathrm{each}}_{}{}_{\;}\mathrm{zone}}{{\mathrm{total}}_{}{}_{\;}\mathrm{Intensity}}}_{}{}_{}{,}_{}{}_{\;}P_{\mathrm{zone}}=\frac{{\mathrm{peak}}_{}{}_{\;}{\mathrm{number}}_{}\;{\mathrm{of}}_{}{}_{\;}{\mathrm{each}}_{}{}_{\;}\mathrm{zone}}{{\mathrm{total}}_{}{}_{\;}{\mathrm{peak}}_{}{}_{\;}\mathrm{number}}$$

### 2.5. Proposing Machine Learning Method

Supervised Learning is a method of machine learning for inferring a function from training data, and supervised learners accurately guess predicted values for a given data from training data [33]. The training data contain the characteristics of the input object in vector format, and the desired result is labeled for each vector. Supervised learning is divided into a predefined classification that predicts one of several possible class labels and a regression that extracts a continuous value from a given function [34].

In order to predict brightness and contrast for better edge detection, we label the collected data using histograms and apply supervised learning. Types of classification methods that produce not continuous results including Support Vector Machine (SVM), K-Nearest Neighbor (KNN), Multilayer Perceptron (MLP), etc.

First, SVM is known as one of the most powerful classification tools [35]. The general concept of SVM is to classify training samples by hyperplane in the space where the samples are mapped. Therefore, SVM only requires training samples close to the class boundary, so high-dimensional data can be processed using a small number of training samples [36]. 

KNN is one of the most basic and simple classification methods. When there is little or no prior knowledge of data distribution, the KNN method is one of the first choices for classification. It is a nonparametric classification system that bypasses the probability density problem [37].

**Figure 6:**
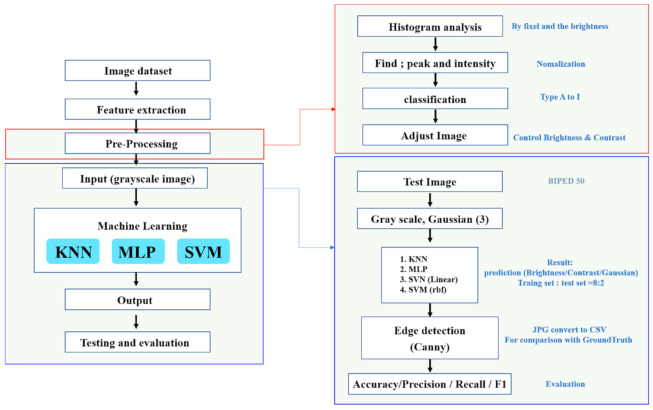
Proposed Framework.

MLP is the most common choice and corresponds to a functional model where the hidden unit is a sigmoid function [38]. These are feed-forward networks where the input flows only in one direction to the output, and each neuron in the layer connects to all neurons in the successive layer, but there is no feedback for the neurons in the previous layer. As far as hidden layers and the number of units are concerned, you should choose a topology that provides optimal performance [39]. We carry out machine learning as shown in Figure 6.

### 2.6. Performance Evaluation

Mean square error (MSE) is the average of the square of the error and it calculates the variance of the data values at the same location between two images. It measures the average difference of pixels in the entire original ground truth image with the edge detection image. Higher MSE means there is a greater difference between the original image and the processed image.

The peak signal-to-noise ratio represents the maximum signal-to-noise ratio and peak signal-to-noise ratio (PSNR) is an objective measurement method to evaluate the degree of change in an image. PSNR is generally expressed in decibel (dB) scale and higher PSNR indicates higher quality [40].

Furthermore, the Structural similarity index measure (SSIM) was not used in the measurement method. Because our method performs edge detection by adjusting the brightness and contrast of the original image. SSIM evaluates how similar the brightness, contrast, and structural differences are compared to the original image. So, it is not suitable for evaluating our image [41].

We perform edge detection of the image applying the canny algorithm to the pre-processed image. Next, we measure the MSE and PSNR between each resulting edge detection image and the ground truth image.

### 2.7. Model Evaluation Method

Describes the metrics used to evaluate the classification performance of a model or pattern in machine learning.

As a performance evaluation index, we selected the following items. First, Precision is the ratio of the actual object edge among those classified as object edges and the ratio of those classified as object edges among those classified as object edges by the model was designated as the Recall value.

Lastly, the F1 score is the harmonic average of Precision and Recall. When the data label is unbalanced, it is possible to accurately evaluate the performance of the model and the performance can be evaluated with a single number.

## 3. Results

**Figure 7:**
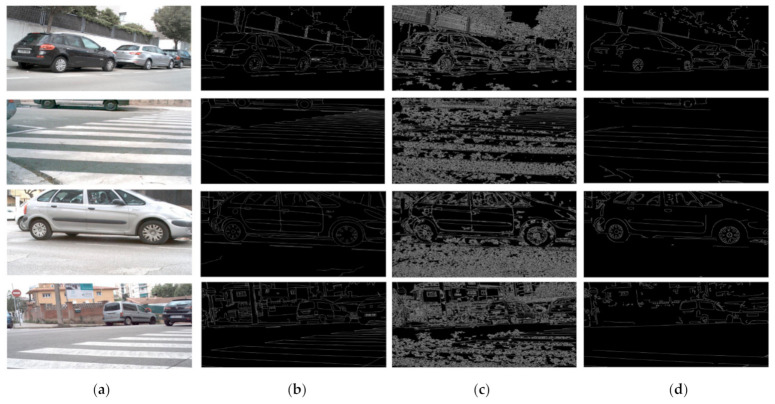
We can see the edge result images without our method (pre-processing about brightness and contrast control) and them with: (**a**) original image; (**b**) Ground Truth; (**c**) Edge detection result with only Canny algorithm; (**d**) Edge detection result with our method.

In the experiment, the most of testing set is categorized in type F, H, E, B therefore we compare F1 score of these types to test the performance of our method comparing original image without pre-processing with pre-processing in BIPED dataset. Not only the scores but also the edge detection result of the image is shown in Figure 7. It can be seen from Figure 7c that only Canny algorithm without pre-processing is too sensitive to noise. Compared with only Canny edge detection, our method maintains meaningful edge by overcoming the noise.

**Figure 8:**
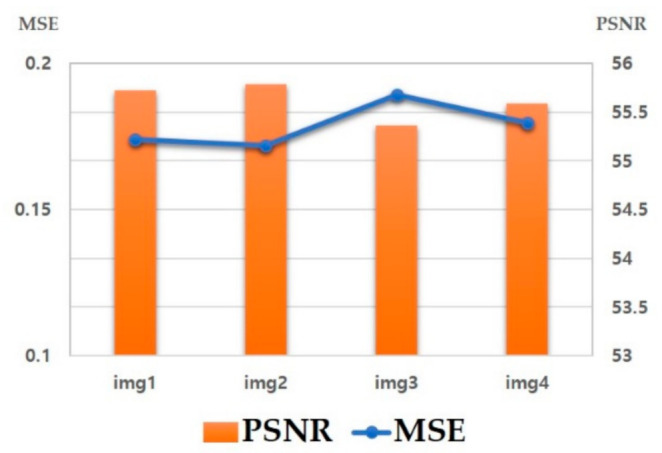
Result of mean square error (MSE), peak signal-to-noise ratio (PSNR) per image.

As shown in Figure 8, the MSE was 0.168 and the PSNR was 55.991 dB. Standard deviation was 0.04 for MSE and 1.05 dB for PSNR and the difference in results between the images was small. Table 2 shows the results of MSE and PSNR according to the edge detection method. It was confirmed that adjusting the brightness and contrast increases the function of edge detection according to the image characteristics through the PSNR value. Furthermore, Table 2 lists the PSNR of the different methods. For the dataset used in each paper, “Rena”, “Baboon”, and “Pepper” were mainly used, and the number of pixel arrays that can affect the value of PSNR and the number of datasets used were entered.

**Figure 9:**
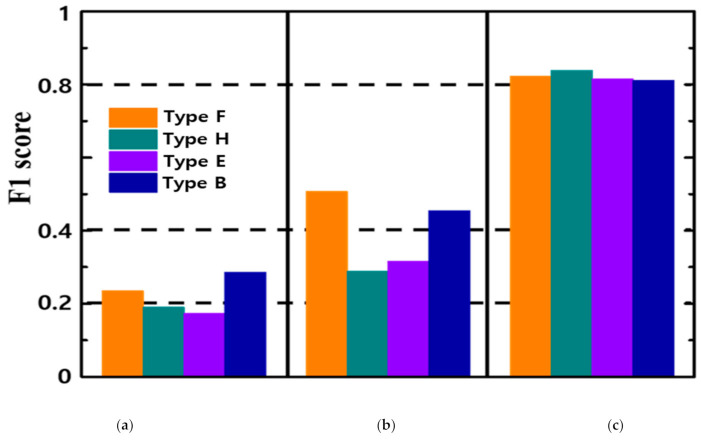
Evaluation result of four images (F1 score): (**a**) Image without pre-processing; (**b**) Image with pre-processing before learning; (**c**) Image with pre-processing after learning.

As shown in Figure 9, our method obtained the best F-measure values in BIPED dataset. It is proved that our method improve performance on F-measure from 0.235 to 0.823. It clearly illustrates the importance of preprocessing task in various illumination image and the performance can be enhanced through learning.

## 4. Discussion

The pre-processing method uses the basic information like brightness and contrast of the image, so you can simply select the characteristics of the data. In addition, if image pre-processing is performed using this method, ISP can find ROI more easily and faster than before. Furthermore, the phenomenon caused by not finding an object, such as flickering of AF seen when the image is bright or the boundary line is ambiguous, will also be reduced. Although testing was conducted with many image samples and data sets, there was a limitation in deriving various information because it was limited to the histogram type used in the data set. Therefore, afterwards, it is necessary to diversify and extract characteristics such as brightness and contrast by securing its own data set. The processing speed of pre-processing takes several minutes to the final step of receiving the image of the dataset, analyzing the histogram, applying the feature, and detecting the edge. In the case of processing speed, the speed can be sufficiently reduced by upgrading the graphic processor unit (GPU). It is necessary to run it on a real board and get the result.

Furthermore, the method we propose is to facilitate edge detection by using the basic information of the image as a pre-process to complement the ISP function of the CMOS image sensor when the brightness is strong or the contrast is low, the image itself appears hazy like a watercolor technique, it is possible to find the object necessary for AWB or AE at the ISP more clearly and easily using pre-processing we suggest. In addition, power consumption or noise can be reduced. In the case of hardware complexity, the method we used is image pre-processing for edge detection. Since the image was processed by the edge detection algorithm after receiving the existing image in the form of a file, it is necessary to consider proceeding the overall process of edge detection using the value input to the CMOS image sensor using a board equipped with an actual processor.

## 5. Conclusions

In this research, we a propose pre-processing method on light control in image with various illumination environments for optimized edge detection with high accuracy. Our method can improve the quality of image by adjusting brightness and contrast, which results in effective edge detection than implementation without light control. So, we see that our edge result achieves the best F-measure. It would be interesting to study further on detection of textures and roughness in images with varying illumination. In addition, the pre-processing we propose can respond more quickly and effectively to the perception of an object by detecting the edge of the image. In particular, it is used for ISP pre-processing so that it can recognize the boundary lines required for operation faster and more accurately, which improves the speed of data processing compared to the existing ISP. It will be useful for autonomous cars, medical information, aviation and defense industries, etc.

## Figures and Tables

**Table 1:** Type by the brightness and contrast.

	I_zone_	I_I_ > 0.5 $\sum I_{\mathrm{II}\sim V}\leq 0.5$	I_V_ > 0.5 $\sum I_{I\sim \mathrm{IV}}\leq 0.5$	Other
P_zone_				
**P_I_ > 0.5** $\sum P_{\mathrm{II}\sim V}\leq 0.5$		A	B	C
**P_V_ > 0.5** $\sum P_{I\sim \mathrm{IV}}\leq 0.5$		D	E	F
Other		G	H	I

**Table 2:** The Comparison with other edge detection methods.

Method	MSE	PSNR	Resolution	Method	MSE	PSNR	Resolution
Proposed	0.168	55.991	1280 × 720	X-OR [42]	0.122	57.240	512 × 512
Robust Wavelet [43]	-	54	512 × 512	IPVD [44]	0.272	53.785	512 × 512
V-bpp Edge-XOR [45]	0.288	53.532	512 × 512	PVD [46]	0.459	52.511	512 × 512
AE_LSB [47]	0.409	52.011	512 × 512	ANFIS [48]	0.454	51.559	-
Improved Hash [49]	-	47.559	-	Hash [50]	-	46.774	512 × 512
weighted vector median filter [51]	24.660	34.210	256 × 256	Fuzzy Edge Detection [52]	51.170	31.040	256 × 256
Fractional Fourier Transform [53]	171.580	25.786	256 × 256	Fuzzy C-means [54]	6714.759	22.708	-
Median Filter [55]	-	18.850	-	Neural Network Approach [56]	-	16.340	-
Novel Wavelet Edge Detection [57]	-	15.670	-	Novel Method [58]	5911.663	10.413	-
Ant Colony Optimization Algorithm [59]	8233.091	8.975	-	D. Poobathy [40]	19567.442	5.216	-
Mouad, M.H.Ali [60]	20073.852	5.127	-	-	-	-	-
